# Effects of Hypoxia on the Immunomodulatory Properties of Adipose Tissue-Derived Mesenchymal Stem cells

**DOI:** 10.3389/fimmu.2013.00203

**Published:** 2013-07-18

**Authors:** Marieke Roemeling-van Rhijn, Fane K. F. Mensah, Sander S. Korevaar, Maarten J. Leijs, Gerjo J. V. M. van Osch, Jan N. M. IJzermans, Michiel G. H. Betjes, Carla C. Baan, Willem Weimar, Martin J. Hoogduijn

**Affiliations:** ^1^Department of Internal Medicine, Erasmus MC, Rotterdam, Netherlands; ^2^Department of Orthopedics, Erasmus MC, Rotterdam, Netherlands; ^3^Department of General Surgery, Erasmus MC, Rotterdam, Netherlands

**Keywords:** mesenchymal stem cells, cell therapy, hypoxia, immune modulation, oxygen level

## Abstract

Adipose tissue-derived mesenchymal stem cells (ASC) are of great interest as a cellular therapeutic agent for regenerative and immunomodulatory purposes. The function of ASC adapts to environmental conditions, such as oxygen tension. Oxygen levels within tissues are typically much lower than under standard culture conditions and ASC used for therapy therefore encounter a change from normoxic to hypoxic conditions. The effect of hypoxia on the regenerative potential of ASC has been investigated in a number of studies. The effect of hypoxia on the immunomodulatory function of ASC, however, remains to be determined. In the present study the effect of hypoxic (1% oxygen) culture conditions on human ASC was examined. ASC showed no signs of toxicity under low oxygen levels and no major immunophenotypical changes were observed, apart from a down regulation of the marker CD105. Oxygen tension had no effect on the proliferation of ASC and colony forming unit efficiency remained the same under 1 and 20% oxygen. Under both oxygen levels ASC were capable of strong upregulation of the immunomodulatory molecules indoleamine 2,3-dioxygenase (IDO) and programed death ligand-1 upon stimulation with IFN-γ and TNF-α, and, in addition, IDO activity as measured by the accumulation of l-kynurenine was not affected under hypoxia. The ability of ASC to inhibit anti-CD3/CD28 stimulated CD4^+^ and CD8^+^ T cell proliferation was not hampered by hypoxia. The results of the present study demonstrate that the immunosuppressive capacity of ASC is maintained under hypoxic conditions. These findings are important for the therapeutic use of ASC and may be applied for the *in vitro* generation of ASC with improved functionality for therapeutic use.

## Introduction

Mesenchymal stem cells (MSC) have emerged as cells with great clinical potential. *In vitro* studies have demonstrated the immunosuppressive and regenerative capacities of MSC. Currently, MSC have been evaluated as a cell therapeutic agent in many medical fields including graft versus host disease, solid organ transplantation, and Crohn’s disease (1–3). MSC can be isolated from a wide range of tissues (4, 5), of which bone marrow is the classical, and most frequently used source. However, bone marrow aspiration is invasive and is accompanied with donor morbidity (6). In contrast, adipose tissue is more accessible, has a higher yield of MSC, and adipose tissue-derived mesenchymal stem cells (ASC) share many properties with their bone marrow derived counterparts (7, 8). Adipose tissue is therefore the favored source of MSC in an increasing number of studies (9–11). To obtain sufficient numbers of MSC for research and certainly clinical application the cells are culture expanded. Culture conditions, however, have striking effects on the phenotype and function of MSC. Advantage of this can be taken by modulating culture conditions in such a way that cells with superior functionality are obtained.

Oxygen concentration is an important environmental factor that affects MSC. While MSC are normally cultured under 20% oxygen tension, tissue-resident MSC face much lower oxygen concentrations. Oxygen tension in adipose tissue, for instance, fluctuates depending on blood flow, but varies typically between 3 and 11% (12). Lower oxygen concentrations can occur in the incidence of injury. It has been demonstrated that hypoxic conditions affect the function of bone marrow derived MSC. Culturing under 1% oxygen reduces MSC senescence while it increases proliferation and maintains the differentiation properties of the cells (13). Similar results have been obtained for MSC derived from adipose tissue and Wharton’s jelly (14–16). This suggests that MSC are triggered by injury-induced hypoxic conditions to expand and eventually differentiate. MSC have also been shown to enhance their angiogenic potential under hypoxia by increasing their secretion of vascular endothelial growth factor (VEGF) and bFGF (17).

Tissue trauma is almost without exception followed by inflammation. Inflammation in its turn is a major activator of the immunosuppressive capacity of MSC (18), which thereby allows regeneration by inhibiting immune activity. It is however unknown whether hypoxia, occurring before the initiation of inflammation, alters the immunosuppressive capacities of MSC. As these capacities are essential for MSC therapy, it is important to evaluate the effect of hypoxia on MSC. Therefore, in the present study we examined the effect of low oxygen concentrations on the phenotype and immunomodulatory properties of human ASC.

## Materials and Methods

### Adipose tissue

Subcutaneous adipose tissue was surgically removed from healthy live kidney donors during the kidney donation procedure after written informed consent, as approved by the Medical Ethical Committee of the Erasmus MC (protocol no. MEC-2006-190). Adipose tissue was collected in essential medium alpha (MEM-α) (Life Technologies, Paisley, UK) supplemented with 100 U/ml penicillin and 10,000 U/ml streptomycin (p/s) and 2 mM l-glutamine (Lonza, Verviers, Belgium).

### ASC isolation

Adipose tissue-derived mesenchymal stem cells were isolated from adipose tissue of five donors as described previously (5, 19). In brief, adipose tissue was mechanically disrupted, enzymatically digested with sterile 0.5 mg/ml collagenase type IV (Sigma-Aldrich, St. Louis, MO, USA) in RPMI-1640 + glutaMAX (Life Technologies) and p/s for 30 min at 37°C. Cells were resuspended in ASC culture medium, consisting of MEM-α with 15% fetal bovine serum (FBS) (Lonza), transferred to a 175 cm^2^ culture flask (Greiner Bio-one, Essen, Germany), and kept at 37°C, 5% CO_2_, 20% O_2_, and 95% humidity. Medium was changed every 3–4 days. When>90% confluent, ASC were detached using 0.05% trypsin-EDTA at 37°C and either directly used for experiments or frozen until usage. ASC were used for experiments between passages 1 and 5.

### Immunophenotyping by flow cytometry

Adipose tissue-derived mesenchymal stem cells were immunophenotyped by flow cytometry after standard culture expansion and after 1 week exposure to hypoxic culture conditions. Subconfluent ASC were trypsinized and washed with FACSFlow (BD Biosciences, San Jose, CA, USA). Cell suspensions were incubated with mouse-anti-human monoclonal antibodies against CD45-PerCP, CD73-PE, CD166-PE, HLA-ABC-APC, HLA-DR-APC-Cy7 (all BD Biosciences), and CD105-FITC (R&D Systems, Abingdon, UK) at room temperature in the absence of light for 15 min. After two washes with FACSFlow, flow cytometric analysis was performed using an eight color FACSCANTO-II with FACSDIVA Software (BD Biosciences) and FlowJo Software (Tree Star, Palo Alto, CA, USA).

### Hypoxic culture conditions

Hypoxic conditions were induced by culture of ASC in 1% O_2_, 5% CO_2_, and 94% N_2_ in a 95% humidified atmosphere. Control cells were kept under normoxic conditions (20% O_2_).

### Lactate measurements

Adipose tissue-derived mesenchymal stem cells were seeded in six-well plates at a concentration of 200,000 cells per well in ASC culture medium and kept under either normoxic or hypoxic conditions. After 3 days, the conditioned medium was collected and frozen at −80°C until usage. A lactate assay kit (BioVision, Milpitas, CA, USA) was used for measurement of lactate levels. In brief, samples were incubated with 50 μl of the Reaction Mix for 30 min at room temperature in the dark. The formed product was measured spectrophotometrically at 570 nm on a Victor^2^ 1420 multilabel plate reader (PerkinElmer, Santa Clara, MA, USA) and corrected for background.

### Measurement of metabolic activity by MTT assay

Adipose tissue-derived mesenchymal stem cells were seeded in 96-well flat bottom plates at a concentration of 3000 cells per well in 200 μl ASC culture medium and kept for 24, 48, or 72 h under normoxic or hypoxic conditions. Five hours prior to the end of the incubation, 20 μl sterile MTT (Sigma-Aldrich) (5 mg MTT/ml dissolved in 1× PBS) was added to the wells. Culture medium was then removed and 100 μl DMSO added to the cells to dissolve formed crystals. Mitochondrial conversion of MTT was determined by absorbance measurements at 550 nm wavelength on a Victor^2^ 1420 multilabel plate reader.

### Colony forming unit assay

Adipose tissue-derived mesenchymal stem cells were seeded at 50 cells per 6 cm diameter culture dishes in quintuple (3.0 cells/cm^2^). After 2 weeks of culture under normoxic or hypoxic conditions, medium was removed, the dishes washed with PBS, and fixed in 70% ethanol. Colonies were stained with 2.3% crystal violet solution (Sigma-Aldrich) for 30 min. Dishes were then washed with tap water and colonies with a diameter of more than 1 mM counted. Colony forming unit (CFU) efficiency was expressed as the percentage of cells capable of forming colonies.

### Proliferation assay

Adipose tissue-derived mesenchymal stem cells were seeded at a density of 1000 cells/cm^2^ in 75 cm^2^ culture flasks and kept under normoxic or hypoxic conditions for 1 week without medium changes. Cells were then detached by trypsinization, counted, and re-seeded. Population doublings were calculated using the formula 2log (number of cells at *t*_d7_/number of cells at *t*_d0_). Cumulative population doublings during five consecutive weeks of culture were plotted.

### Quantitative mRNA expression

Adipose tissue-derived mesenchymal stem cells were seeded in six-well plates at a concentration of 1 × 10^5^ per well in the presence or absence of 20 ng/ml TNFα and 50 ng/ml IFNγ and kept under normoxic or hypoxic conditions. After 6, 24, or 72 h, RNA*later* (Life Technologies) was added to the cells. RNA was isolated and 500 ng used for cDNA synthesis as described previously (20). Gene expression was determined by real-time RT-PCR using universal PCR master mix (Life Technologies) and Assays-on-demand for hypoxia induced factor 1α (HIF1-α) (Hs00153153.m1), HIF2-α (Hs1026149.m1), VEGF (Hs00173626.m1), indoleamine 2,3-dioxygenase (IDO) (Hs00158627.m1), PDL-1 (Hs00204257.m1), TGFβ1 (Hs00171257.m1), IL10 (Hs00174086.m1), IL6 (Hs00174131.m1), and CXCL10 (Hs00171042.m1) (all Applied Biosciences, Foster City, CA, USA) on an ABI PRISM 7700 sequence detector (Applied Biosystems). As housekeeping gene expression fluctuates under hypoxia, data was expressed as relative copy number of the PCR products per 500 ng RNA. Relative copy number was calculated using the formula 2^(40-Ct value)^.

### IDO activity measurements

Adipose tissue-derived mesenchymal stem cells were cultured for 24 or 72 h with or without 20 ng/ml TNF-α and 50 ng/ml IFN-γ under normoxic or hypoxic conditions. The tryptophan metabolic activity of IDO was determined by measurement of l-kynurenine in the conditioned medium of five ASC cultures. Thirty percent of trichloroacetic acid was added to the samples at a 1:3 ratio and after 30 min incubation at 50°C the samples were centrifuged at 12,000 rpm for 5 min. Supernatants were then diluted 1:1 in Ehrlich reagent [200 mg 4-dimethylaminobenzaldehyde (Sigma, St. Louis, MO, USA) in 10 ml of glacial acetic acid] in duplicate in a 96-wells flat bottom plate and absorbance was determined at 490 nm using a multilabel plate reader (VersaMax™, Molecular Devices, Sunnyvale, CA, USA). l-Kynurenine (Sigma, St. Louis, MO, USA) diluted in unconditioned medium was used as standard.

### PBMC isolation

Peripheral Blood Mononuclear Cells (PBMC) were collected from buffy coats of healthy blood bank donors. PBMC were isolated by density gradient centrifugation using Ficoll Isopaque (δ = 1.077, Amersham, Uppsala, Sweden) and frozen −135°C until use.

### Anti-CD3/CD28 lymphocyte stimulation assay

Peripheral blood mononuclear cells were labeled using the PKH26 Red Fluorescent Cell Linker Kit (Sigma-Aldrich) and stimulated with anti-CD3 antibody (0.5 μl/5 × 10^5^ cells), anti-CD28 antibody (0.5 μl/5 × 10^5^ cells), and a goat-anti-mouse antibody (1 μl/5 × 10^5^ cells) for cross-linking (all BD Biosciences). PBMC were seeded in round-bottom 96-well plates at 5 × 10^4^ cells per well and ASC added at 1:2.5, 1:5, 1:10, and 1:20 ratios in MEM-α with p/s and 10% heat inactivated (HI)-human serum. After 3 days of incubation under normoxic or hypoxic conditions, PBMC were collected, washed twice with FACSFlow (BD Biosciences), and incubated with monoclonal antibodies against TCR-FITC (Serotec, Oxford, UK), CD3-AmCyan, CD4-Pacific Blue, CD8-PerCP (all BD Biosciences) for 15 min at room temperature in the absence of light. After two washes with FACSFlow, flow cytometric analysis was performed using an eight color FACSCANTO-II with FACSDIVA Software (BD Biosciences).

### Statistical analysis

Data were statistically analyzed using Mann Whitney, and Kruskal–Wallis statistical tests. *p* < 0.05 was considered significant.

## Results

### ASC identity

In several previous studies we have demonstrated that the adherent cells of the stromal vascular fraction of human adipose tissue are ASC with properties similar to bone marrow MSC (5, 19, 21). Thus ASC have a spindle-shaped fibroblastic morphology in culture, have a CD45^−^CD73^+^CD105^+^CD166^+^HLAclass I^+^ immunophenotype and can differentiate in adipogenic and osteogenic lineages (19) (data not shown).

### Culture of ASC under hypoxic conditions

**Figure 1:**
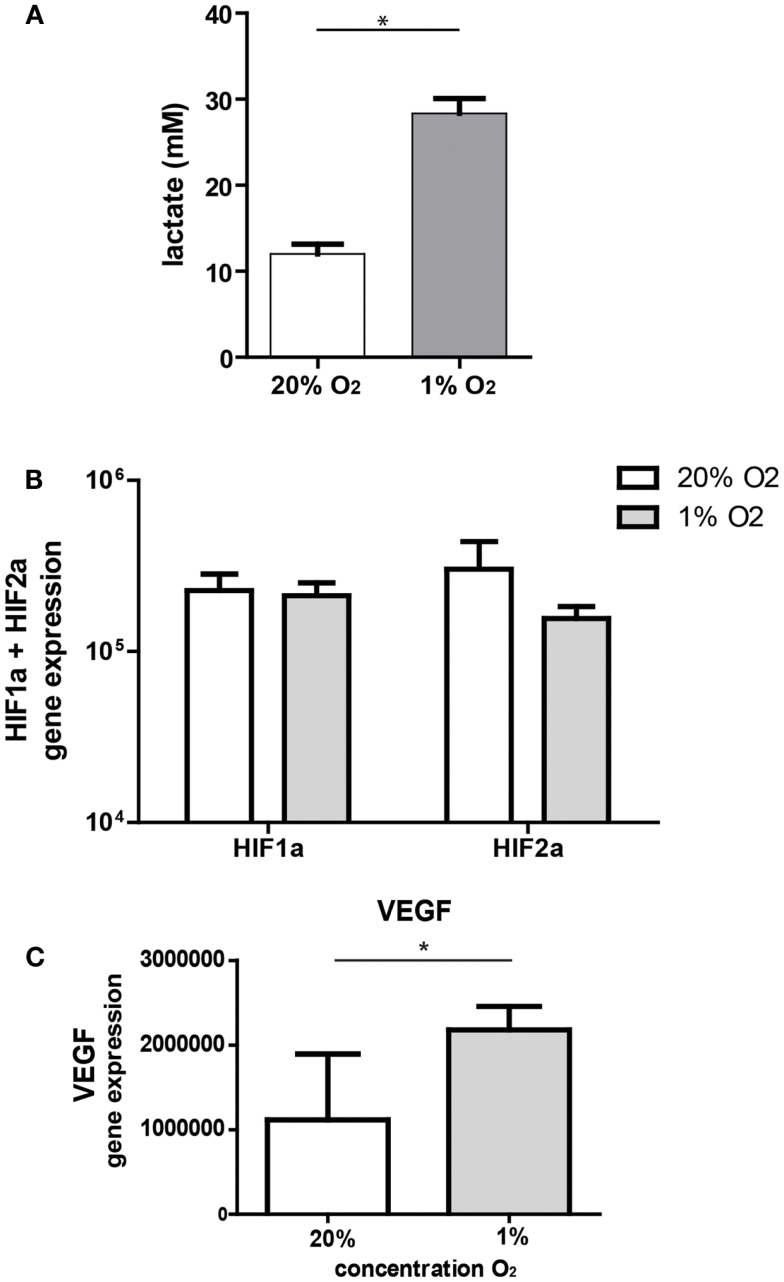
**(A)** Lactate production by ASC. ASC were cultured under 20 or 1% O_2_ for 3 days and medium collected for lactate measurements. Mean with SEM of three experiments is shown. **(B)** Effect of hypoxia on gene expression of hypoxia-inducible genes; mRNA expression of HIF1-α and HIF2-α by ASC after 24 h culture under normoxic (20% O_2_) and hypoxic (1% O_2_) conditions. Mean with SD of four different experiments is shown. **(C)** mRNA expression of VEGF, downstream of HIF1-α under normoxia and hypoxia. Mean with SD of four different ASC cultures, *Indicates*p* < 0.05.

Under hypoxic conditions, cells are forced to switch to anaerobic metabolism and will produce lactate. In our model, ASC significantly increased lactate concentrations in the culture medium after 3 days of culture under hypoxic conditions (28.3 mM, range 25–31 versus 12 mM, range 10–14, under normoxic conditions) (Figure 1A). Culture of ASC under hypoxic conditions (1% O_2_) for 24 h had no effect on mRNA expression levels of hypoxia-inducible factor 1-α (HIF1-α) and HIF2-α (Figure 1B). As hypoxia stabilizes HIF protein, we examined whether gene expression downstream of HIF was affected by hypoxia in ASC. Expression of VEGF downstream of HIF1-α was significantly increased under hypoxia by nearly a factor two (Figure 1C).

### Effect of hypoxia on ASC characteristics

**Figure 2:**
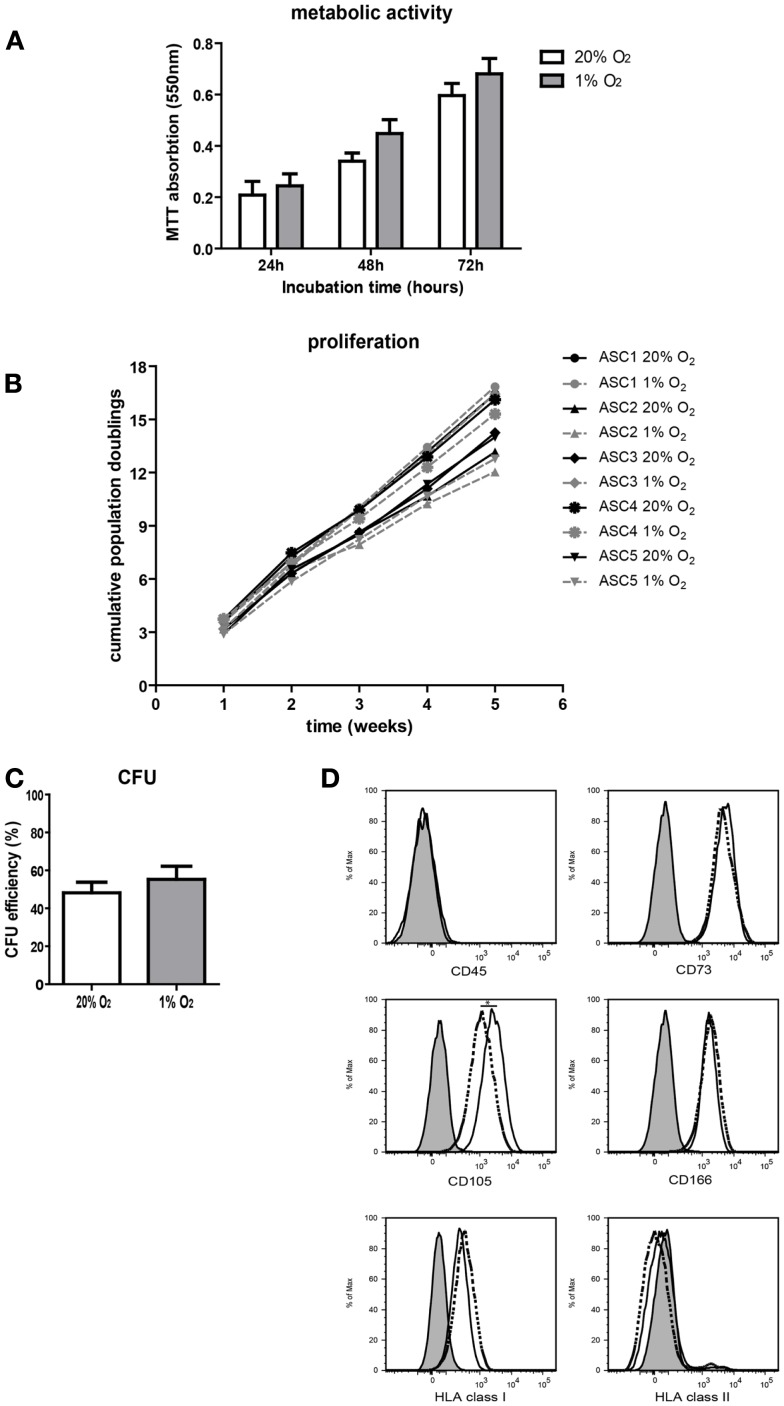
**Effect of hypoxia on ASC characteristics**. **(A)** ASC metabolic activity measured by MTT assay. Mean with SEM of three different ASC cultures shown. **(B)** Cumulative population doublings of ASC cultured under normoxic and hypoxic conditions. **(C)** CFU efficiency of ASC. Mean with SEM of four different ASC cultures shown. **(D)** Immunophenotype of ASC after culturing for 10 days under normoxic and hypoxic conditions. Filled histogram; unstained ASC, solid line; ASC cultured under 20% O_2_, dotted line; ASC cultured under 1% O_2_. *Indicates*p* < 0.05.

To study the effect of hypoxia on ASC characteristics, metabolic activity, survival, growth, and proliferation of ASC was examined. Culture of ASC under 1% O_2_ for 24 h up to 72 h had no effect on ASC metabolic activity and there was no evidence for loss of cell viability, as measured by the mitochondrial reduction of MTT to formazan (Figure 2A). There was a similar increase in the conversion of MTT under normoxic and hypoxic conditions at 24 h up to 72 h, which reflects the similar increase in cellular metabolic activity under both conditions. Parallel culture of ASC under normoxic and hypoxic conditions for five consecutive weeks demonstrated no difference in population doubling times, which were 2.6 days (range 1.9–3.1), under normoxia and 2.5 days (range 1.9–5.0) under hypoxia. Therefore, cumulative population doublings were the same under both oxygen tensions (Figure 2B). Furthermore, there was no difference in the CFU capacity of ASC cultured under 20 and 1% O_2_ (Figure 2C), suggesting oxygen tension does not affect the stemness of ASC. Finally, the immunophenotype of ASC was determined after 10 days of culture under normoxic or hypoxic conditions. Under both conditions, the ASC immunophenotype was CD45^−^CD73^+^CD105^+^CD166^+^HLA-I^+^HLA-II^−^ (Figure 2D). The expression of CD105 was, however, significantly lower in ASC cultured under hypoxic conditions (MFI 2530) than in ASC cultured under normoxia (MFI 4215).

### Effect of hypoxia on the expression of immunomodulatory genes by ASC and IDO activity

**Figure 3:**
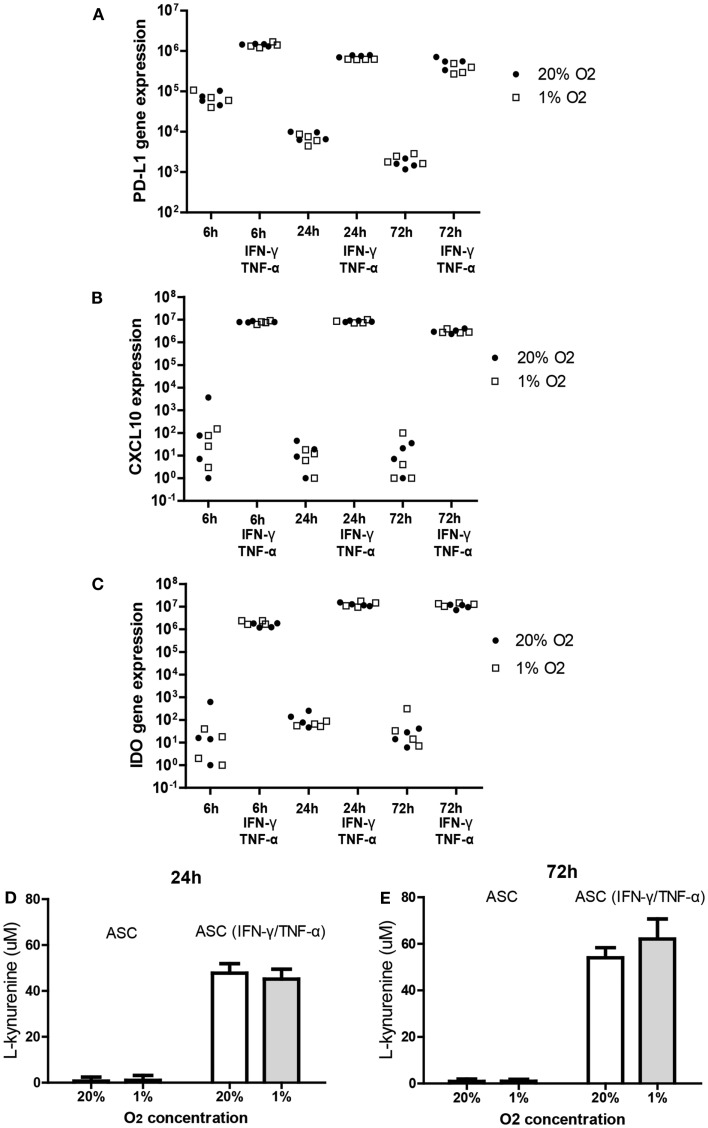
**Effect of hypoxia on the induction of PD-L1 (A), CXCL-10 (B), and IDO (C); mRNA expression in ASC after IFN-γ/TNF-α stimulation**. ASC were cultured under 20 or 1% O_2_ for 6, 24, or 72 h with or without 50 ng/ml IFN-γ and 20 ng/ml TNF-α. Every data point represents a distinct ASC culture (*n* = 4 different ASC cultures). **(D,E)** IDO activity determined by accumulation of l-kynurenine in ASC conditioned medium in the absence and presence of TNF and IFN [24 h **(D)** and 72 h **(E)**]. Means of five ASC cultures with SEM shown.

It is known that the immunomodulatory capacity of ASC is induced under inflammatory conditions. This was confirmed in the present study by showing that treatment of ASC with IFN-γ and TNF-α for 6, 24, and 72 h induced a strong increase in the mRNA expression of immunomodulatory programed death ligand-1 (PD-L1) (10^2^-fold), CXCL10 (10^6^-fold), and IDO (10^5^-fold). Under hypoxic conditions, ASC maintained the capacity to induce the expression of IDO, PD-L1, and CXCL10 in response to IFN-γ and TNF-α to a similar extent as under normoxic concentrations (Figures 3A–C).

To determine whether hypoxia affected the tryptophan depleting activity of IDO in ASC we measured concentrations of l-kynurenine, the breakdown product of tryptophan, in conditioned medium of ASC cultured for 24 or 72 h with or without TNF-α and IFN-γ under 20 or 1% oxygen. Culture with TNF-α and IFN-γ strongly increased l-kynurenine levels at 24 and 72 h (Figures 3D,E). Hypoxia did not affect l-kynurenine levels indicating preserved IDO activity under hypoxic conditions.

Furthermore, TGF-β, associated with the immunomodulatory capacity of ASC, was expressed by ASC under resting conditions and at similar levels upon stimulation with IFN-γ and TNF-α. Hypoxia did not affect TGF-β expression (data not shown). IL10 was not expressed by ASC under any of the conditions tested (data not shown). Pro-inflammatory IL6 was expressed by ASC, and its expression did not change under low oxygen conditions (data not shown). These data suggest that ASC maintain their capacity to express immunomodulatory factors and respond to inflammatory conditions by activating their immunomodulatory apparatus under hypoxic conditions.

### Immunosuppressive capacities of ASC under hypoxia

**Figure 4:**
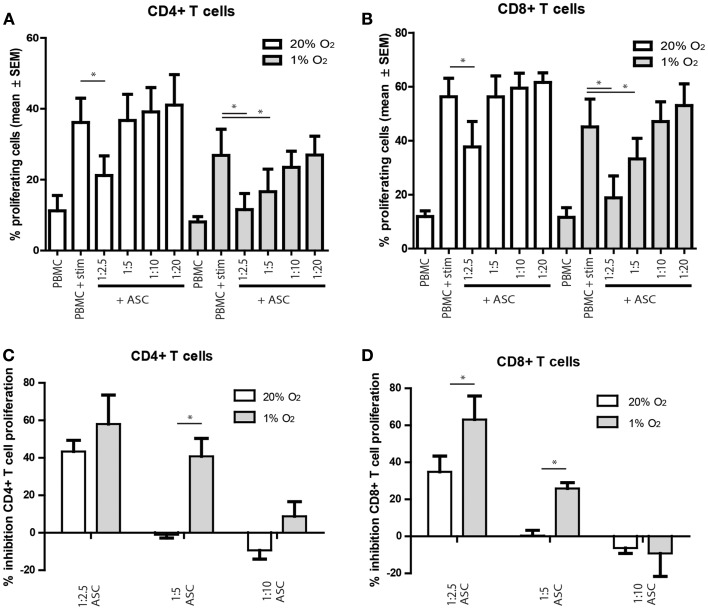
**(A–D)** PBMC were labeled with PKH and stimulated with anti-CD3/CD28 antibodies. On day 3, the proliferation of CD4^+^ and CD8^+^ T cells was analyzed by flow cytometry. **(A,B)** percentage proliferating cells shown. **(C,D)** percentage inhibition of proliferation shown, compared to the condition without ASC. Mean with SEM of three experiments with different ASC cultures shown, *p-value < 0.05.

To study whether hypoxia influences the *in vitro* immunosuppressive capacities of ASC, ASC were added to anti-CD3 and anti-CD28 stimulated PKH-labeled allogeneic PBMC. The proliferation of CD4^+^ and CD8^+^ T cells tended to be lower under hypoxia than under normoxia, although the difference was not significant. Under normoxia, there was a significant inhibition of CD4^+^ and CD8^+^ T cell proliferation at ASC: PBMC ratios of 1:2.5 (Figures 4A,B). Under hypoxia, inhibition of T cell proliferation was more profound as T cell proliferation was significantly inhibited till ASC:PBMC ratios up to 1:5 (Figures 4A,B). Direct comparison of the T cell inhibition by ASC under normoxia and hypoxia demonstrated that inhibition of CD4^+^ and CD8^+^ T cell proliferation was significantly higher at a 1:5 under 1% oxygen level (Figures 4C,D).

## Discussion

Adipose tissue-derived mesenchymal stem cells possess an assortment of properties that make them suitable for regenerative and immunomodulatory applications. The properties of ASC are affected by changing environmental conditions that induce ASC to adapt a particular function. Oxygen tension is one of these environmental factors. Oxygen tension in tissue fluctuates between 3 and 11% (22) and may be lower in case of trauma, and such changes may affect the function of ASC. In the present study, we cultured ASC under 20 and 1% oxygen to examine whether oxygen tension would affect the *in vitro* properties of ASC.

In the present study we found that ASC were resistant to hypoxic conditions and detected no signs of toxicity or decreased CFU efficiency under low oxygen concentrations. There is some controversy on the effect of hypoxia on the proliferation of bone marrow and ASC, with some studies reporting an inhibitory effect of low oxygen levels on proliferation (23– 25), others reporting increased proliferation (26), while we found that ASC proliferation was not affected over at least five passages. There are a number of factors that may influence the outcome of studies, such as the percentage of oxygen used to generate hypoxic conditions with some studies using 1% oxygen and others up to 5%. Furthermore, the composition of the cell culture medium, in particular the use of serum, and the species of ASC used will affect outcomes. In the present study, the immunophenotype of ASC was only moderately affected by hypoxia, which is in line with earlier studies (24, 25). In our hands, only the expression of CD105 (endoglin) was consistently down regulated under low oxygen tension. This is quite surprisingly, as CD105 is known to be up regulated under hypoxia in endothelial cells (27). The consequence of the down regulation of CD105, which is an adhesive molecule, and part of the TGFβ receptor complex remains to be determined.

In the current study, we further found that a hypoxic pre-conditioning regimen does not hamper the immunosuppressive properties of ASC. The immunosuppressive function of ASC is induced by inflammatory cytokines such as IFN-γ and TNF-α (18, 28). These cytokines stimulate the expression of anti-proliferative IDO and of the inhibitory co-stimulatory molecule PD-L1. The induction of these proteins and the activity of IDO were not affected by hypoxic conditions, indicating that the immunosuppressive machinery of ASC is maintained at low oxygen concentrations. In addition, the strongly enhanced expression of the chemokine CXCL10 under inflammatory conditions was not affected by hypoxia, suggesting that the chemoattractive properties of ASC for immune cells are preserved under low oxygen concentrations.

Subsequently, we found that culture under 1% O_2_ did not hinder the suppressive effects of ASC on T cell proliferation. The percentage of inhibition of T cells by ASC was even increased under hypoxia. Thus although ASC under hypoxia were phenotypically indifferent from ASC under normoxic conditions, they had a more profound effect on T cell proliferation. One explanation could be that hypoxic conditions shift the balance between T cell proliferation and the inhibitory effect of ASC. Thus while T cells are affected in their proliferation by hypoxia, as demonstrated earlier (29) and shown in the present study, ASC maintain their suppressive capacity. It is possible that under hypoxic conditions, T cells are more sensitive to low tryptophan concentrations induced by IDO expression in ASC or by PD-L1 induced inhibition of proliferation. ASC therefore have a relatively larger impact on inflammatory conditions and are more effective in inhibiting immune responses under hypoxic conditions, such as in case of trauma, than under normoxic conditions. Alternatively, the enhanced efficacy of ASC to inhibit T cell proliferation under hypoxia may be explained by the fact that ASC employ additional mechanisms of immunomodulation under hypoxic conditions that were not analyzed in the present study.

In summary, our data indicates that a reduction of oxygen tension up to at least 1% does not hamper the immunomodulatory therapeutic efficiency of ASC. This is of relevance as for most clinical purposes, the immunosuppressive capacities of ASC are essential. Assertion of these capacities under hypoxia is crucial as ASC will encounter hypoxic conditions when administered for most, if not all, immunomodulatory applications. Subsequently, as there is evidence that hypoxic pre-conditioning enhances the regenerative potential of ASC (17, 30), maintenance of immunosuppressive capacities under hypoxia is needed when hypoxia pre-conditioning will be used for regenerative application. The present study shows that in these situations, the immunomodulatory capacity of ASC is preserved.

## Conflict of Interest Statement

The authors declare that the research was conducted in the absence of any commercial or financial relationships that could be construed as a potential conflict of interest.
